# Tumor biomechanical stiffness by magnetic resonance elastography predicts surgical outcomes and identifies biomarkers in vestibular schwannoma and meningioma

**DOI:** 10.1038/s41598-024-64597-1

**Published:** 2024-06-24

**Authors:** Bailey H. Duhon, Kristin Thompson, Melanie Fisher, Vivian F. Kaul, Han TN. Nguyen, Michael S. Harris, Varun Varadarajan, Oliver F. Adunka, Daniel M. Prevedello, Arunark Kolipaka, Yin Ren

**Affiliations:** 1 Division of Otology, Neurotology, and Cranial Base Surgery, Department of Otolaryngology-Head and Neck Surgery, The Ohio State University Wexner Medical Center, Columbus, OH USA; 2 Otology, Neurotology, and Lateral Skull Base Surgery Program, Department of Otorhinolaryngology-Head and Neck Surgery, McGovern Medical School at the University of Texas at Houston, Houston, TX USA; 3 Department of Radiology, The Ohio State University Wexner Medical Center, Columbus, OH USA; 4 Department of Otolaryngology and Communication Sciences, Medical College of Wisconsin, Milwaukee, WI USA; 5 Department of Neurosurgery, The Ohio State University Wexner Medical Center, Columbus, OH USA

**Keywords:** Vestibular schwannoma, Meningioma, Stiffness, Magnetic resonance elastography, MRI, Surgical oncology, Translational research, Tumour biomarkers, Magnetic resonance imaging

## Abstract

Variations in the biomechanical stiffness of brain tumors can not only influence the difficulty of surgical resection but also impact postoperative outcomes. In a prospective, single-blinded study, we utilize pre-operative magnetic resonance elastography (MRE) to predict the stiffness of intracranial tumors intraoperatively and assess the impact of increased tumor stiffness on clinical outcomes following microsurgical resection of vestibular schwannomas (VS) and meningiomas. MRE measurements significantly correlated with intraoperative tumor stiffness and baseline hearing status of VS patients. Additionally, MRE stiffness was elevated in patients that underwent sub-total tumor resection compared to gross total resection and those with worse postoperative facial nerve function. Furthermore, we identify tumor microenvironment biomarkers of increased stiffness, including αSMA + myogenic fibroblasts, CD163 + macrophages, and HABP (hyaluronic acid binding protein). In a human VS cell line, a dose-dependent upregulation of *HAS1-3*, enzymes responsible for hyaluronan synthesis, was observed following stimulation with TNFα, a proinflammatory cytokine present in VS. Taken together, MRE is an accurate, non-invasive predictor of tumor stiffness in VS and meningiomas. VS with increased stiffness portends worse preoperative hearing and poorer postoperative outcomes. Moreover, inflammation-mediated hyaluronan deposition may lead to increased stiffness.

## Introduction

Biomechanical stiffness, a visco-elastic property related to tumor consistency, is associated with worse clinical outcomes in both benign and malignant tumors^1–3^. This association has been investigated in brain tumors and hepatocellular carcinoma, where stiffer tumors required longer operating time, led to higher rates of sub-total resections and worse postoperative outcomes such as cranial nerve palsy. Nonetheless, the role of tumor stiffness on clinical outcomes has yet to be investigated in patients undergoing surgical resection of vestibular schwannoma (VS), the most common cerebellopontine angle (CPA) neoplasm arising from the vestibulocochlear nerve (CN VIII)^4^ VS is associated with significant morbidities such as deafness and vertigo due to the tumor’s proximity to eloquent brainstem structures^4^. Microsurgical resection remains the only curative treatment for large or symptomatic tumors with significant brainstem compression as there are no FDA-approved pharmacotherapies. While gross total tumor resection is the goal, the extent of resection and postoperative morbidities vary significantly. During surgery, some tumors are soft resembling that of fatty tissue, while others are extremely firm. These stiffer VS are less amenable to central debulking, requiring more brain retraction, more piecemeal dissections and higher ultrasonic aspirator settings, increasing the risk of iatrogenic injury from manipulation at the tumor-brain or nerve interfaces.

Unfortunately, surgeons currently have little-to-no *a-priori* knowledge regarding the tumor’s biomechanical stiffness prior to surgery, nor do they have a “stiffness map” to anticipate and help navigate anatomical areas where difficulties could be encountered during tumor dissection. This effectively leaves surgeons blind until a craniotomy has been performed. Current imaging modalities such as T2-weighted MRI do not directly measure tissue stiffness. Ultrasound elastography can measure tissue stiffness of organs not encased in bone but is not applicable for intracranial lesions in the preoperative setting^5^. Tools such as atomic force microscopy (AFM) are not currently adapted for clinical use^6^. By contrast, magnetic resonance elastography (MRE) is a noninvasive modality that measures the visco-elastic properties of liver, brain, breast, aorta and heart^1,7,8^. Mechanical cyclic vibrations are induced in the regions of interest to obtain wave data in the phase of MR images and processed into stiffness maps. Recent literature has shown that MRE can predict stiffness of VS and meningioma (MN)^9–11^. However, these studies failed to evaluate postoperative outcomes nor examine the molecular determinants of stiffness. Therefore, there is a critical unmet need for accurate pre-operative assessment of the biomechanical properties of intracranial tumors that could guide surgeons by predicting the intraoperative experience.

We hypothesize that increased tumor stiffness is characterized by dysregulated fibrotic deposition during tumor extracellular matrix (ECM) remodeling. Here, we show that patients with stiffer CPA tumors as determined by MRE pre-operatively have worse outcomes than soft tumors following surgery, including higher rates of facial nerve palsy and increased incidence of subtotal tumor resection. Finally, we provided molecular insights into the development of stiff VS.

## Methods

Detailed methods are available in the Supplementary Methods section.

### Patient enrollment and study design

The study was approved by the Institutional Review Board at The Ohio State University Wexner Medical Center, and written informed consent was obtained from all patients (IRB #2012H0007, IRB#1994H0241). Patients with sporadic VS and MN were prospectively enrolled between March 2016 to January 2023, and underwent MRE imaging within 72-h prior to planned tumor resection. The surgical team was single blinded to the MRE data at the time of surgery. The decision to perform operative intervention was based on a multidisciplinary discussion considering clinical factors including tumor size and configuration based on MR imaging, hearing status in the ipsilateral ear, onset and severity of symptoms that are attributable to the tumor, medical comorbidities, and the preference of the patient and their families. All experiments were performed in accordance with the relevant guidelines and regulations.

### Magnetic resonance elastography

All MRE imaging was performed using a 3 T MRI scanner (Tim Trio and Prisma, Siemens Healthcare, Erlangen, Germany). Axial slices were obtained using a spin-echo echo planar imaging (SE-EPI) MRE sequence. T2-weighted and T1-weighted fluid-attenuated inversion recovery (FLAIR) images were acquired to clearly identify the tumor-brain interface. 60 Hz vibrations were introduced through a soft pillow-like driver that is placed underneath the head in a birdcage coil (brain coil). The setup used for brain MRE utilizes an active driver that is placed outside scanner room and vibrations are induced using a soft pillow-like driver underneath the subject's head. Imaging parameters included: FOV = 256 × 256 mm, matrix size = 256 × 256 mm, TR/TE = 1800/43.4 ms, slice thickness = 3 mm, ~ 16 slices based on tumor coverage, MRE phase offsets = 4. Motion encoding gradient of 60 Hz was applied separately in the x, y and z directions to encode in-plane and through plane displacement fields. Total scan time was approximately 6 min.

### Elastogram DI processing

MRE images were masked to delineate the brain and a curl processing was performed to remove longitudinal component of motion. Additionally, a directional filter was applied to remove the reflected waves. Finally, direct inversion (DI) with a Laplacian of Gaussian filter was performed to compute a weighted stiffness map, or elastogram.

### Intraoperative assessment of stiffness

To validate the utility of MRE to quantitatively assess tumor stiffness, a board-certified, fellowship-trained otologist/neurotologist and skull-base neurosurgeon collected intraoperative tumor stiffness measurements during microsurgical resections for a subset of patients (n = 10). Each surgeon worked independently to measure intraoperative stiffness using a quantitative survey (Supplementary Table 1). The intratumoral firmness was graded from 1 to 5 (5 being most stiff) based on the surgeon's consensus impressions and maximum ultrasonic aspirator settings required to debulk the tumor. To limit potential bias, the surgeons recorded the settings for the ultrasonic aspirator at two different intratumoral locations, adjacent to the brainstem and adjacent to the cerebellum, to measure tumor consistency. The remainder of the tumors (n = 10) were resected by the same surgical team, however did not include prospectively collected intraoperative stiffness.

### Tumor processing

Clinical diagnoses were made by a board-certified neuropathologist according to 2016 WHO guidelines. Tissue samples were fixed in 10% formalin, embedded in paraffin, sectioned at 5 microns and analyzed by immunohistochemical staining.

### Clinical data analysis

Demographic and clinical data were queried from chart review. Tumor volume was calculated on the most recent preoperative MRI with the modified ellipsoid formula: 4/3× π × ½ (W× L× H), where W, L, H represent the width, length, and height of the tumor in all three axes^12^. Tumor diameter was noted as the largest cross-sectional diameter on axial T1-weighted, contrast-enhanced MRI slices in the CPA (whether anteroposterior (AP), mediolateral (ML), or craniocaudal (CC)), including the internal auditory canal (IAC) component for the ML measurement. Audiometric data included bone-conduction pure-tone average at 500, 1000, 2000, and 4000 Hz and word recognition scores (WRS). Serviceable hearing was classified as a post-operative WRS of greater than 50%. Gross total tumor resection (GTR), defined as complete tumor excision with no visible tumor left in situ, was attempted in all cases. Near total resection (NTR) was defined as having a residual tumor measuring less than 5 × 5 × 2 mm, which was typically left along the facial nerve (FN) or brainstem. If more tumor was left behind, this was defined as a subtotal resection (STR). The decision to perform less-than-total tumor resection was made intraoperatively based on factors including the relative morbidity of attempting total tumor removal and the integrity of the FN. In cases where the tumor capsule was adherent or inseparable from the brain or when complete tumor removal would disrupt the anatomic continuity of the FN, a NTR or STR was performed. Postoperatively, FN function was graded as good (House-Brackmann [HB] grade I–III) and poor (HB grade higher than III) within 2 days postoperatively (immediate) and again at greater than 6 months postoperatively (long-term). For any discrepancies in FN function between physician reports, the most senior operating surgeon’s determination was used. This was most often the attending otologist/neurotologist.

## Results

**Figure 1:**
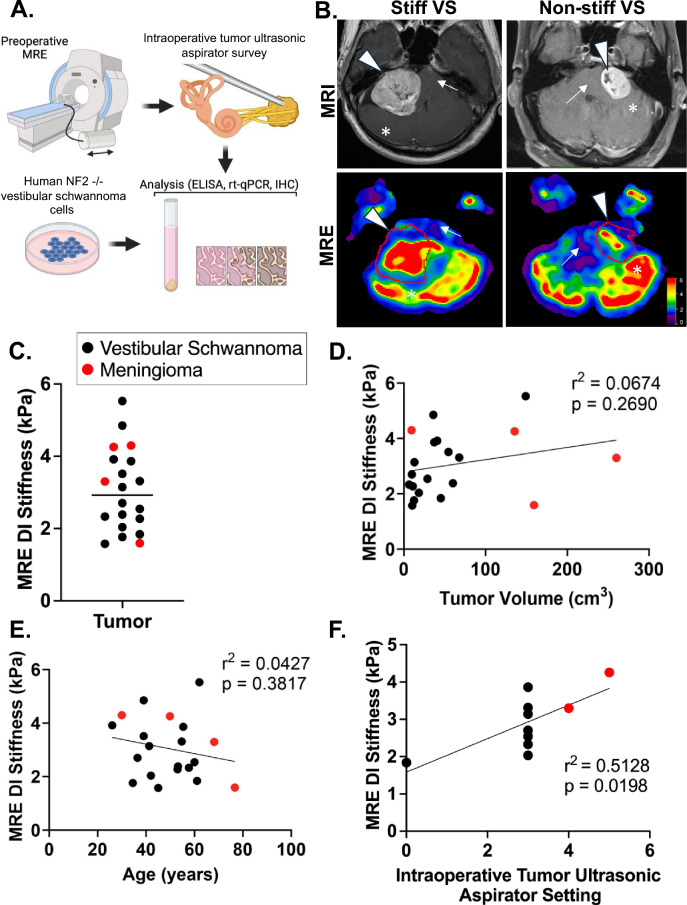
Magnetic resonance elastography predicts stiffness in posterior cranial fossa tumors (**A**) Schematic of patient workflow. Prospectively enrolled patients diagnosed with either vestibular schwannomas or meningiomas scheduled for surgical resection underwent MRE 24–72 h prior to surgery. During surgery, the intraoperative experience and tumor consistency was recorded independently by the surgical team blinded to MRE results. Following surgery, tumors were collected and analyzed. (**B**) Representative T1-MRI (top row) and elastograms (bottom row) of a stiff (left column) and non-stiff (right column) vestibular schwannoma. Arrowhead denotes tumor; arrow denotes brainstem; * denotes cerebellum. An elastogram color scale bar is shown. (**C**) Preoperative DI MRE stiffness across the entire patient cohort. The black line represents the mean stiffness at 3.05 kPa. (**D**). MRE stiffness does not significantly correlate with tumor volume, *p* = *0.269* by analysis of the Pearson correlation coefficient. (**E**). MRE stiffness did not correlate with patient age, *p* = *0.388* by analysis of the Pearson correlation coefficient. (**F**) Preoperative MRE stiffness correlates with intraoperative survey measurements of tumor consistency determined by ultrasonic aspirator settings (higher settings indicate greater force), *p* = *0.020* by analysis of the Pearson correlation coefficient.

A total of 20 patients with VS or MN were enrolled (70% male, mean age 49 ± 13 years) (Fig. 1A and Table 1). Representative T1-based contrast-enhanced MRI images and their corresponding MR elastograms are shown (Fig. 1B). The average DI stiffness based on MRE was 3.1 kPa (range; 1.6 to 5.5 kPa) (Fig. 1C). Nearly half of the VS were macrocystic (n = 9, 56%), while all MNs were solid tumors. All patients had preoperative audiograms with mean pure tone average thresholds (PTA) of 37 dB HL (range, 10 to 81 dB HL) and word discrimination score (WRS) in quiet of 60% (range, 0 to 100%) in the ipsilateral ear. Of the eleven patients (55%) that underwent attempted hearing preservation surgery (retrosigmoid craniotomy), five (45%) had some degree of hearing preserved postoperatively, with an average postoperative PTA of 51 dB HL (range, 25 to 81 dB HL) and mean postoperative WRS of 72% (range, 24 to 100%). The remaining 9 patients (45%) underwent translabyrinthine craniotomy and did not have postoperative audiograms.

**Table 1:** Demographics and clinical outcomes of patients with posterior fossa tumors.

	All (n = 20)	VS (n = 16)	MN (n = 4)
Age (years)	49 ± 13	48 ± 11	56 ± 20
Male (n, %)	14 (70%)	12 (75%)	2 (50%)
MRE Stiffness (kPa, range)	3.05 (1.58—5.53)	2.97 (1.58—5.53)	3.36 (1.59—4.30)
PTA (dB HL)	36.2	37.0	33.3
WRS (%)	60	55.3	65.0
Tumor Characteristics			
Maximum diameter (cm, range)	3.12 (1.80—5.80)	2.77 (1.80—4.60)	4.55 (1.98—5.80)
Volume (cm^3^, range)	58.2 (6.4 – 260.1)	37.5 (6.4 – 149.1)	141.1 (9.4 – 260.1)
Cystic (n, %)	9 (45%)	9 (56%)	0 (0%)
Surgical Approach			
Retrosigmoid (n, %)	11 (55%)	7 (44%)	4 (100%)
Translabyrinthine (n, %)	9 (45%)	9 (56%)	0
Extent of resection			
Gross-/Near-total resection (n, %)	14 (70%)	16 (75%)	2 (50%)
Sub-total resection (n, %)	6 (30%)	4 (25%)	2 (50%)
Postoperative Outcomes			
FN grade, immediate (n, %)			
Good (HB I—III)	18 (90%)	14 (88%)	4 (100%)
Poor (HB > III)	2 (10%)	2 (12%)	0
FN grade, long-term (n, %)			
Good (HB I—III)	18 (90%)	14 (88%)	4 (100%)
Poor (HB > III)	2 (10%)	2 (12%)	0

Postoperatively, fourteen patients (70%) underwent either GTR/NTR of their tumor and six (30%) underwent STR. When stratified by tumor size, all tumors less than 3.0 cm in maximum diameter (n = 10 of 10) underwent GTR/NTR, whereas 60% of tumors greater than 3.0 cm in diameter (n = 6 of 10) underwent STR. The average diameter of tumors that underwent STR was 4.1 cm (range, 3.0 to 5.6 cm). In the immediate post-operative period and at long-term follow-up (6–12 months postoperatively), nearly all patients (91.6%, n = 18 of 20 VS and n = 4 of 4 MN) demonstrated good FN function (HB grade I–III).

### Magnetic resonance elastography predicts overall tumor stiffness

Next, to validate MRE’s predictive capability for intraoperative stiffness, the preoperative MRE stiffness measurements were compared to the operating surgeon’s intraoperative assessment of stiffness (Supplementary Table 1). Surveys were completed for 10 subjects (8 VS and 2 MN), and MRE stiffness significantly correlated with tumor stiffness collected using the intraoperative survey instrument (r^2^ = 0.51, *p* = *0.019*) (Fig. 1F). On univariate analysis, MRE stiffness was predictive of stiff and non-stiff tumors determined from intraoperative assessments (*p* = *0.011*).

### Stiff (firm) vs non-stiff (soft) tumors

To examine if stiffness is correlated with other demographic or tumor characteristics, patients were classified into two groups, those with stiffness above the mean (Stiff or firm, n = 10) and those below the mean MRE-derived stiffness of 3.1 kPa (Non-stiff or soft, n = 10). Demographic comparison of the two groups were shown in Table 2. As expected, stiffness significantly varied between stiff and non-stiff groups (3.99 vs. 2.10 kPa, *p* < *0.001*), whereas age (*p* = *0.373),* and gender (*p* = *0.314)* did not (Table 2). Age, gender, and tumor volume similarly did not correlate with MRE stiffness (Fig. 1D, Fig. 1E & Supplementary Figure S1). Tumor diameter, however, was significantly higher in stiff tumors (3.5 vs. 2.7 cm, *p* = *0.043)* (Table 2). Stiffer tumors were also comprised more of solid tumors as compared to non-stiff tumors, where cystic tumors predominated (*p* = *0.035*). There were no significant differences in the proportion of VS in either group (*p* = *0.291*). Moreover, the patients with stiff and non-stiff tumors underwent similar types of surgical approaches, confirming the single-blinded nature of the study as the surgical approach was not influenced by preoperative MRE findings.

**Table 2:** Comparison between stiff versus non-stiff posterior cranial fossa tumors.

	Stiff (n = 10)	Non-Stiff (n = 10)	p-value
Age (years)	47 ± 14	52 ± 13	0.373
Male (n, %)	6 (60%)	8 (80%)	0.314
MRE Stiffness (kPa, range)	3.99 (3.14 – 5.53)	2.10 (1.58 – 2.70)	< 0.001
Tumor Characteristics			
Maximum diameter (cm, range)	3.54 (2.21 – 5.80)	2.71 (1.80 – 5.60)	0.043
Volume (cm^3^, range)	80.3 (9.4 – 260.1)	36.1 (6.4 – 159.2)	0.053
Cystic (n, %)	2 (20%)	7 (70%)	0.035
Histology			
Vestibular Schwannoma (n, %)	7 (70%)	9 (90%)	0.291
Meningioma (n, %)	3 (30%)	1 (10%)	
Surgical approach			
Retrosigmoid (n, %)	5 (50%)	6 (60%)	0.999
Translabyrinthine (n, %)	5 (50%)	4 (40%)	

### Tumor stiffness is related to worse patient outcomes

**Figure 2:**
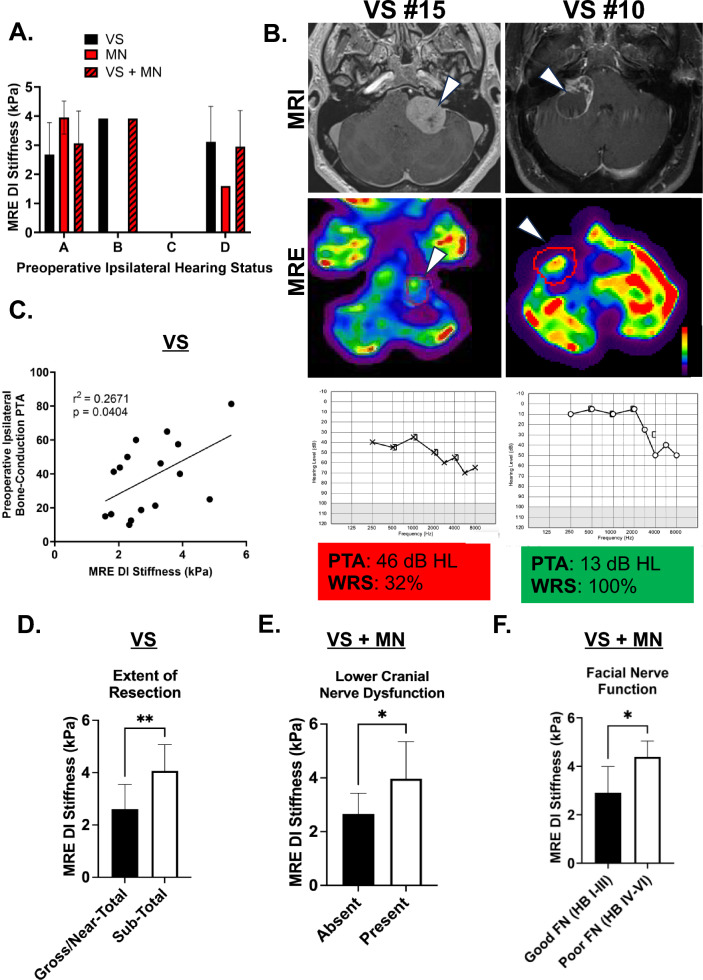
Posterior fossa tumor stiffness is elevated in cases with worse preoperative hearing and poorer postoperative outcomes. (**A**) Preoperative MRE DI stiffness is not significantly different in patients with worse preoperative hearing based on bone-conduction pure-tone average and word recognition scoring. Audiometric data is classified into grades (**A**–**D**) based on the 1995 AAO-HNS grading system, with grade D being the worst. (**B**) Representative T-1 MRI (top row), elastograms (second row), and audiograms (third row) for a patient with poor hearing and a stiff tumor (left column, 3.3 kPa) and a patient with good hearing with a soft tumor of similar size (right column, 2.4 kPa). Tumor volumes were similar (68 cm^3^ for the stiff tumor and 61 cm^3^ for the non-stiff tumor). Pure tone average (PTA) and word recognition scores in quiet (WRS) are noted below the respective audiograms. Arrowhead denotes tumor. Color bar denotes stiffness from 0 to 6 kPa. (**C**) In vestibular schwannomas, MRE stiffness is positively correlated with baseline bone-conduction PTA (db HL). (**D**) MRE stiffness is significantly elevated in VS that required a sub-total resection compared to those amenable to a gross or near-total resection. ** *p* < 0.01 by Mann-Whitney *U*-test. (**E**) MRE stiffness was higher in patients with lower cranial nerve dysfunction. * *p* < 0.05 by Mann-Whitney *U*-test. (**F**) MRE stiffness was elevated in patients with poor long-term facial nerve function (HB > III) compared to those with good facial nerve function (HB I-III). * *p* < 0.05 by Mann-Whitney *U*-test.

We next examined if MRE stiffness may be predictive of baseline hearing status and postoperative outcomes. Out of the 20 patients who had completed audiograms preoperatively, stiffness did not vary significantly across AAO-HNS hearing classes (Fig. 2A). Representative T-1 weighted MRI and MRE elastograms of VS patients with good hearing and poor hearing are shown in Fig. 2B. In VS, there was a significant correlation between MRE stiffness and baseline hearing measured by bone-conduction PTA (r^2^ = 0.27, *p* = *0.040*) (Fig. 2C). There was no significant difference in preoperative WRS in quiet (58% vs 56%, *p* = *0.448*) (Supplementary Figure S2). Stiffness was significantly elevated in STR VS compared to those that underwent GTR/NTR (3.72 kPa vs 2.36 kPa, *p* = *0.010*) (Fig. 2D).

In the immediate postoperative period, MRE stiffness correlated with FN function (r^2^ = 0.36, *p* = *0.001*). Patients with postoperative lower cranial nerve (CN IX–XII) dysfunction had stiffer tumors than those without (3.97 kPa vs. 2.65 kPa, *p* = *0.020*) (Fig. 2E). Notably, 30% (n = 3 of 10) of the patients with stiff tumors experienced temporary dysphagia following surgery, with one developing aspiration pneumonia and another requiring a percutaneous endoscopic gastrotomy tube, while there were no postsurgical complications associated with aspiration in patients with non-stiff tumors (*p* = *0.305*). At long-term follow-up (median follow-up time 61 months, range 6 to 81 months), patients with sustained poor FN function (HB > III) had significantly elevated stiffness as compared to tumors with good FN function (4.38 vs 2.62 kPa, *p* = *0.047*) (Fig. 2F). Taken together, our data indicates that patients with stiffer posterior fossa tumors tend to experience a higher incidence of STR and postoperative morbidities such as facial palsy, dysphonia and dysphagia.

With tissue stiffness associated with poor outcomes following tumor extirpation, we next analyzed if other clinical factors (namely tumor size, age and surgical approach) impacted baseline hearing (Supplementary Fig. 3A), FN function and the extent of tumor resection. Tumor volume did not correlate with baseline hearing status measured by pure tone thresholds (*p* = *0.211*, Supplementary Fig. 3B) or long-term FN function (38.4 cm^3^ in HB > III vs. 37.4 cm^3^ in HB ≤ III, *p* = *0.350*, Supplementary Fig. 3D). Additionally, neither age and nor surgical approach varied significantly between tumors with good and poor FN function (*p* > *0.050* respectively, Supplementary Fig. 3C). Comparing patients who underwent STR to those undergoing GTR/NTR, tumor volume was larger in the STR group (78.0 cm^3^ vs 24.0 cm^3^, *p* = *0.004*, Supplementary Figure S3F) and a higher proportion had solid rather than cystic tumors (83% vs. 43%, *p* = *0.157*) (Supplementary Fig. 3E).

### Vestibular schwannomas vs meningiomas

We next analyzed if the histological subtype of the tumor could contribute to differences in clinical outcomes. MNs were slightly stiffer than VSs although this did not reach statistical significance (3.36 kPa vs 2.97 kPa, *p* = *0.279*) (Table 1), likely due to the small number of MN in our cohort. Regarding lower cranial nerve function, 100% of MN experienced temporary dysfunction compared to only 12.5% of VS (*p* = *0.003*). This is likely due to the MN having an average craniocaudal diameter of 3.8 cm compared to 2.3 cm in VS, with MNs extending more inferiorly towards the lower cranial nerves. At long-term follow-up, FN outcomes in VS were concordant with the entire combined cohort, demonstrating increased stiffness in tumors with poor FN outcomes (HB > III) (4.38 kPa vs. 2.46 kPa in VS with good FN function, *p* = *0.033*), while all MNs demonstrated complete (100%) recovery of FN function. Overall, VS stiffness correlated with worse preoperative hearing, worse FN function, and extent of resection. Moreover, stiffer MN are more likely to negatively impact speech and swallowing functions following surgery.

### Molecular characterization of stiff VS

**Figure 3:**
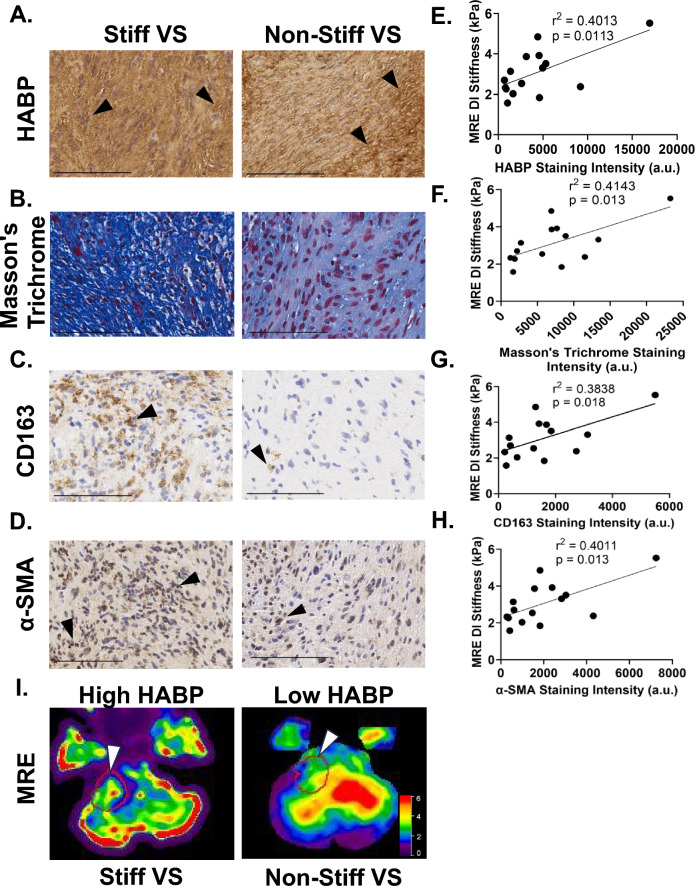
Histopathological features correlate with MRE predictive stiffness. Representative IHC images of stiff (left column) and non-stiff (right column) vestibular schwannoma tumors stained for hyaluronan (HABP)(**A**), Masson’s Trichrome (**B**), M2 Macrophages (CD163) (**C**), and activated fibroblasts (α-SMA) (**D**). Scale bar represents 100 µm. Positive staining were denoted by arrowheads. (**E**–**H**) Correlation between MRE DI stiffness and staining intensities of hyaluronan (HABP, (**E**), Masson’s Trichrome (**F**), M2 macrophages (CD163, (**G**), and activated fibroblasts (α-SMA, (**H**). All *p* < 0.05 by analysis of Pearson correlation coefficient. (**I**) Representative elastograms of a stiff VS tumor (left column, 4.9 kPa) with high HABP staining intensity versus a soft VS tumor (right column, 2.5 kPa) with low HABP staining intensity. The red line and white arrowhead detail the outline of the VS tumor determined by T-1 MRI.

With increased stiffness associated with worse surgical outcomes, we sought to understand the molecular characteristics of VS with increased stiffness. Therefore, we were interested to determine if ECM and cellular components of the TME were altered in stiffer VS, and first investigated the relative amount of Antoni A vs. Antoni B areas in VS. Histological analysis revealed no significant differences in the amount of Antoni A versus B areas between the stiff and non-stiff cohort (Supplementary Figure S4). We further characterized the abundance of ECM components in the tumor parenchyma by immunohistochemistry (Fig. 3). The intensity of Masson’s Trichrome, which stains overall collagen, correlated with stiffness (r^2^ = 0.414, *p* = *0.013*) (Fig. 3B,F). Hyaluronan (hyaluronic acid binding protein, HABP) is a glycosaminoglycan with diverse functions implicated in immune and stromal cell regulation, tumor cell proliferation, and the development of solid stress within the TME^13,14^. HABP demonstrated a moderate/weak positive correlation with tumor stiffness (r^2^ = 0.401, *p* = *0.011*) ((Fig. 3A,E). However, CD44, a cell surface receptor for hyaluronan, did not (r^2^ = 0.105, *p* = *0.239*) (Supplementary Figure S5). The amount of tumor infiltrating M2 macrophages (CD163 +) also statistically significantly, but moderately/weakly, correlated with stiffness (r^2^ = 0.384, *p* = *0.018*) (Fig. 3C,G). Alpha-smooth muscle actin (α-SMA) intensity, denoting activated myogenic fibroblasts, similarly correlated with preoperative MRE stiffness (r^2^ = 0.401, *p* = *0.013*) (Fig. 3D,H). Together, this suggests that fibroblast activation and macrophage infiltration, along with collagen and hyaluronan deposition, are enriched in stiff VS (Fig. 3I).

### Hyaluronan synthesis in vestibular schwannoma cells

**Figure 4:**
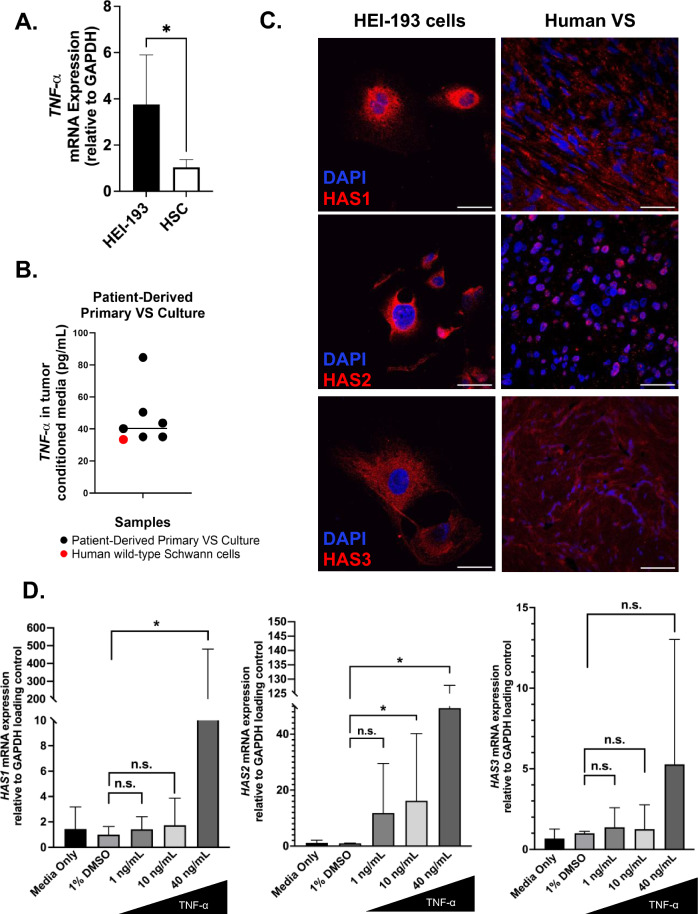
Hyaluronan synthase activity is upregulated in vestibular schwannomas. (**A**) *TNFα* mRNA expression is upregulated in NF2 -/- schwannomatosis cells (HEI-193) versus wild-type human Schwann cells (HSC). *p < 0.05 by Mann Whitney U-test. (**B**) TNF-α is also actively secreted by human primary vestibular schwannoma cells (black dots) at greater levels than Schwann cell controls (red dot). (**C**) Representative images of HAS1 (top), HAS2 (middle), and HAS3 (bottom) expression in HEI-193 cells (left column) and in archived, formalin-fixed paraffin-embedded vestibular schwannomas (right column). Red, HAS enzymes; Blue, DAPI. The scale bar represents 50 µm (left column) and 100 µm (right column). (**D**) Relative expression of *HAS1-3* mRNA normalized to *GAPDH* following TNF-alpha stimulation in HEI-193 cells. Data from three independent experiments are shown as a mean plus standard deviation. *p < 0.05, n.s., not significant, by one-way ANOVA with post-hoc Bonferroni correction.

With HABP demonstrating correlation with MRE stiffness, we sought to understand how hyaluronic acid might be overexpressed in the TME of stiff tumors. TNF-α, a pro-inflammatory cytokine, is secreted by both VS cells and VS-associated macrophages^15^. Additionally, application of VS secretions rich in TNF-α led to cochlear spiral ganglion nerve damage^16,17^. We next sought to investigate whether hyaluronan expression is influenced by TNF-α. We first confirmed the expression of TNF-α in an established human schwannoma cell line (HEI-193) and primary VS cultures established from freshly resected tumor specimens. *TNF-α* mRNA expression was over three-fold higher in the VS cell lines compared to wild-type Schwann cells (Fig. 4A). TNF-α was also actively secreted in primary VS cultures (Mean 44.3 pg/mL, n = 6) (Fig. 4B). Both HEI-193 cells and VS tissue express hyaluronan synthases (HAS1-3) by immunofluorescence, the enzymes responsible for hyaluronan synthesis (Fig. 4C). When HEI-193 cells were exposed to recombinant TNF-α, upregulation of *HAS1, HAS2 and HAS3* mRNA expression was observed (Fig. 4D and Supplementary Figure S6). Taken together, we have demonstrated that TNF-α is actively secreted by schwannoma cells which may then upregulate *HAS* expression and modulate hyaluronan deposition.

## Discussion

Utilizing MRE, we demonstrate that biomechanical properties of VS and MN, common benign neoplasms of the skull base, can be ascertained pre-operatively. Furthermore, tumor stiffness correlated with preoperative hearing and was predictive of clinical outcomes following surgery. Additionally, tumor stiffness may be influenced by the abundance of hyaluronan which may be regulated by proinflammatory cytokines in the TME.

Our initial validation demonstrates that MRE provides an accurate quantitative assessment of the consistency of intracranial neoplasms, as corroborated by other studies^9–11,18–20^. Previous investigations focused primarily on utilizing MRE to predict the tumor consistency of MN not specific to the posterior fossa or CPA, measured by surgeon’s assessments and standardized tools such as a durometer, but have a limited analysis of VS^9–11,18,21^. Moreover, these studies have failed to consider *how* MRE stiffness measurements could actually impact clinical outcomes following surgery or provide insights into *why* stiffer tumors develop^9–11,18^. In contrast, our study aimed to provide insight into the *how* and *why* questions concerning MRE’s clinical utility in tumors specific to the CPA, including VS. We found tumor stiffness was not altered by demographic variables such as age and sex, but is influenced by factors such as tumor size, growth rate and the presence of macrocystic degeneration. Specifically, larger VS tend to exhibit higher stiffness on MRE. This could be explained by both pathological desmoplasia during tumor progression and alterations to the nearby structures by large, stiff tumors. Many desmoplastic tumors demonstrate altered ECM remodeling during progression that leads to increased fibrosis and stiffness^22,23^. Tumor growth in the CPA may be restricted due to the anatomic confines by the petrous temporal bone and limited distensibility of posterior fossa structures. A stiffer tumor may compress and degrade nearby structures to a greater extent, creating a more hypoxic and inflammatory microenvironment, leading to further immune cell infiltration and non-cystic tumor growth^24^. This theory is supported by studies demonstrating widening of the IAC with firm VS^25^. Finally, we demonstrate that cystic VS were softer (non-stiff), likely due to large compressible fluid components as compared to stiff tumors. In addition, overexpression of MMPs in cystic VS, as demonstrated by our earlier findings^26^, may also further degrade the ECM components responsible for stiffness, making cystic tumors softer.

While tumors become stiff as they progress, the relationship between tumor stiffness, tumor volume and hearing status is an area of active debate^25,27^. Hearing loss is a predominant symptom of patients with VS, but the rate of hearing deterioration varies significantly^4^. We demonstrate that stiffness may be involved in preoperative hearing loss in patients with VS. As vestibular schwannomas progress, the tumor escapes the bony confines of the IAC towards the CPA^28^, potentially stretching fibers of the vestibulocochlear nerve. A stiffer tumor could impart higher mechanical shear force on the cochlear nerve fibers irrespective of tumor size, leading to neurodegeneration and ultimately hearing loss. Our data showing tumor stiffness, not volume, weakly correlated with baseline hearing supports this hypothesis, however the translatability of this data is limited by the small number of patients. Furthermore, other studies have proposed additional mechanisms of tumor-associated sensorineural hearing loss including ototoxic tumor secretions or damage to the labyrinthine blood supply^16,17^. Moreover, stiffer tumors may have a more inflammatory and cochleotoxic secretory profile. Consistent with our observations, others have found that MN of the IAC do not impact hearing in temporal bone studies^29^. MN does not originate from the IAC and usually displaces the cranial nerves posteriorly, which is shorter in distance than displacing anteriorly as seen in VS.

Tumor stiffness significantly impacts the intraoperative experience and tumor resectability^1–3^. In hepatocellular carcinoma, higher MRE stiffness correlated with increased operative blood loss and poorer outcomes such as venous thrombosis and liver failure^1^. Similarly, in MNs, stiff and fibrous tumors required longer operating times, increased likelihood of STRs and higher incidence of cranial nerve palsy^2,3^. These findings are corroborated by our study demonstrating vestibular schwannomas not amenable to GTR/NTR exhibited higher stiffness values. These stiff VS may require more piecemeal dissection and greater manipulation of the tumor at the tumor-nerve interface as discussed previously. In some cases, a STR was required to avoid permanent damage to cranial nerves^30^. Moreover, our patients who had stiffer tumors had extended hospital stays and a higher incidence of postoperative complications such as aspiration pneumonia and dysphagia. While not statistically significant, these findings may be critical during preoperative counseling of potential surgical risks and complications.

To our knowledge, our study is the first to correlate preoperatively measured stiffness with cranial nerve outcomes. Stiffness was significantly elevated in patients with poor FN function. While most patients with VS, and all those with MN, recovered FN function in the long-term, two patients who remained HB V and VI had very stiff tumors (mean stiffness of 4.38 kPa). Large CPA tumors extend toward the jugular foramen and can therefore impact lower cranial nerve function. We postulate that the reduced distensibility of these stiff tumors could lead to a greater impact especially when tumors are large. Future studies should include larger cohorts where patients can be further stratified based on tumor volume.

VS progression is characterized by the proliferation of non-myelinating injury-like Schwann cells and infiltration of tumor-associated macrophages (TAMs), with both cell types demonstrating enriched ECM remodeling gene clusters^24,31^. These cellular components of the VS TME release cytokines that induce excessive production of collagen and ECM components by activated fibroblasts^15,32,33^. Additionally, Schwann cell production of endopeptidases, especially matrix metalloproteases (MMPs), act to degrade the ECM proteins as supported by our findings demonstrating upregulated MMP expression in adherent and cystic VS^26^. Taken together, rapid and continuous remodeling of the tumor ECM could lead to increased deposition of misaligned collagen fibers, increased cross-linking between cells and matrix components, and increased hyaluronan synthesis that has been associated with mechanical changes and development of stiffness^13,23,34–36^. In the current study, we demonstrated a statistically significant, yet moderate/weak, correlation between both collagen and hyaluronan deposition with tumor stiffness. Interestingly, CD44, the receptor for hyaluronan, did not correlate with MRE stiffness, suggesting that stiff tumor cells may downregulate CD44 over time, losing their ability to monitor hyaluronan deposition. In VS, Schwann cells take on an injury-like state and demonstrate upregulated secretion of cytokines including TNF-α, TGF-β, IL-1β, and IL-6^31,37^, which activate fibroblasts that subsequently secrete structural components (collagen), adhesion proteins (laminin and fibronectin), and hyaluronan^35,38^. In addition to ECM production, myogenic fibroblasts (α-SMA +) may contract fibrillar ECM components, increasing tension through mechanical stretching and providing mechanical cues for further TAM infiltration^39^. This is corroborated by our findings of elevated numbers of activated α-SMA + fibroblasts in stiff tumors and elevated levels of TNF-α in primary VS cultures. While the correlation analysis revealed statistical significance in all ECM/TME metrics, there is only a moderate correlation coefficient for most analyses. Therefore, these results demonstrate preliminary findings from a small cohort of tumors, with broader implications that motivate future studies.

Additionally, infiltrated TAMs play an important role in the immune regulation of the VS TME^31^. Our findings demonstrate that M2 macrophage infiltration is weakly correlated to stiffness, possibly playing a role in fibroblast activation^15^. Alterations in the tumor mechanics signaled through mechanotransduction pathways may also modulate the proliferation and secretion profiles of tumor cells and cancer-associated fibroblasts^40,41^. Taken together, we demonstrate that the number of activated fibroblasts and infiltrated TAMs is increased in stiff VS and may lead to dysregulated deposition of collagen and hyaluronan. The importance of hyaluronan in modulating mechanical stiffness has been demonstrated in inflammatory processes due, in part, to hyaluronan’s hydrated nature theorized to lead to micro-edema at the site of inflammation^13^. In addition, hyaluronan and its synthesis is well characterized for its role in tumor cell invasion and growth behavior^42,43^. We sought to understand if cytokines in the VS TME could lead to increased hyaluronan synthesis and therefore increased stiffness^36^. When exposed to TNF-α, a pro-inflammatory cytokine secreted by VS^15–17,37^, *HAS1* and *HAS2* were significantly upregulated. Results from this study motivate future investigations into repurposing therapeutics that neutralize TNF-α (i.e. infliximab, adalimumab) to decrease hyaluronan deposition to slow the development of stiff VS^44^. Further TME-associated molecules implicated in the development of stiffness in VS should be explored as potential targets that may be amenable to small molecule and antibody-based therapies. Moreover, additional studies are being performed to investigate how hyaluronan and collagen affect the proliferation of VS both in vitro and in vivo models.

This study has several limitations. MRE was limited to tumors larger than 1 cm^3^ due to the limited spatial resolution. VS that are completely intracanalicular are not included due to the inability to accurately distinguish tumor from brain during MRE processing. Quantification of stiffness intraoperatively is subjected to inter-/intra-observer biases due to the semi-quantitative nature of the survey instrument. In the future, ex vivo stiffness measurements of resected tumor tissue using AFM may aid in confirming the intraoperative assessments. Additionally, tumor aggressiveness is likely multifactorial, although the larger volume may lend towards worse clinical outcomes. Finally, the immunohistochemical analysis of these tumors is preliminary, demonstrating only modest correlation among the studied ECM/TME components and MRE stiffness.

In conclusion, our study demonstrated that biomechanical stiffness in tumors of the CPA, namely VS and MN, as measured by MRE preoperatively, correlated with baseline hearing status, intraoperative experience and postoperative outcomes. Tumor stiffness is elevated in patients who underwent STR and those with poor postoperative FN and lower cranial nerve function. Elevated hyaluronan content is a potential mediator of increased stiffness and may be induced by TNF-α. Overall, MRE offers unique diagnostic and prognostic information that could aid in surgical planning, patient and provider counseling, and surgical outcome improvement.

### Supplementary Information

Supplementary Information.

## Data Availability

The data will be made available upon reasonable request. Please submit your request to the corresponding author (AK and YR).
