# Supplementary material for: Tumor biomechanical stiffness by magnetic resonance elastography predicts surgical outcomes and identifies biomarkers in vestibular schwannoma and meningioma
Source: Sci Rep. 2024 Jun 24;14:14561. doi: 10.1038/s41598-024-64597-1 (PMC11196577; doi:10.1038/s41598-024-64597-1)
Supplement: Supplementary file 1 — Supplementary Information. [file 41598_2024_64597_MOESM1_ESM.docx]

**Supplementary Tables**

**Supplementary Table 1:** Intraoperative quantitative survey

| **Quantitative Intraoperative Survey** | | | | | |
| --- | --- | --- | --- | --- | --- |
| **Consistency**  Please mark all that apply. | | | | | |
| **Not Applicable** | **Liquid** | **Suctionable** | **Resectable at low ultrasonic aspirator setting** | **Resectable at high ultrasonic aspirator setting “rock hard”** | **Unresectable** |
|  | **1** | **2** | **3** | **4** | **5** |
| **Zone**  Please mark all that apply. | | | | | |
| **Not Applicable** | **Root Entry Zone (REZ)** | | **Brainstem** | | **Cerebellum** |
|  | **1** | | **2** | | **3** |

**Supplementary Figure Legends**

**Supplementary Figure S1**: MRE stiffness does not vary between patient sex.

**Supplementary Figure S2**: Preoperative ipsilateral word recognition scoring (WRS) in a quiet environment did not correlate with MRE stiffness in our cohort. *p>*0.05 by analysis of Pearson correlation coefficient. Black dots represent vestibular schwannomas and red dots represent meningiomas.

**Supplementary Figure S3:** Preoperative ipsilateral bone-conduction pure tone average (PTA) does not correlate with patient age (A) or tumor volume (B), all *p>*0.05 by analysis of Pearson correlation coefficient. Patient age (C) or tumor volume (D) does not differ in patients with good facial nerve function (HB I-III) compared to poor FN function (HB > III), n.s., non-significant by Mann-Whitney *U*-test. Age (E) does not vary between patients that underwent sub-total versus near-/gross-total resection, n.s., non-significant by Mann-Whitney *U-*test. However, tumor volume (F) is significantly greater in patients who underwent sub-total resection compared to near-/gross-total resection, ***p<*0.01 by Mann-Whitney *U-*test.

**Supplementary Figure S4**: Representative images of H&E staining of stiff vestibular schwannoma (left column, mean stiffness of 3.89 kPa) and non-stiff vestibular schwannoma (right column, mean stiffness of 2.31 kPa) demonstrating no gross difference in the amount of Antoni A versus Antoni B tissue. Scale bar represents 100 µm. VS# represents tumor identification number.

**Supplementary Figure S5:** A) Representative image of a stiff (left) and non-stiff (right) vestibular schwannoma stained for CD44, a receptor for hyaluronan. B) Graphical representation demonstrating that CD44 does not correlate with MRE stiffness, *p>*0.05 by analysis of Pearson correlation co-efficient.

**Supplementary Figure S6**: **Cell viability assay.** Graphical representation of the MTT assay for cell viability performed on the HEI-193 cells. The viability of cells was negatively impacted (< 80% viability) at doses of TNF-α greater than 40 ng/mL. Cell viability was normalized to the viability of HEI-193 cells in DMEM only with no addition of TNF-α (media-only). **p<0.01, n.s., non-significant, by one-way ANOVA with Bonferroni correction.

**Supplementary Methods**

***Immunohistochemistry***

Formalin-fixed, paraffin-embedded tumor slides (sectioned at 4 microns) were baked at 60℃ for 30 minutes, deparaffinized, underwent heat-induced epitope retrieval. Next, endogenous peroxidase activity was quenched with 1% hydrogen peroxide and subsequently blocked with animal-free blocker (Vector Labs, Newark, CA). Immunohistochemical staining (IHC) was performed with: Hyaluronan (HA, 1:300, GeneTex), CD44 (1:200 Dako Clone: DF1485), CD68 (1:7000 Dako Clone: KP-1), CD163 (1:900 Leica Clone: 10D6), Alpha-SMA (1:600 Dako Clone: 1A4). For each antibody, appropriate negative controls were utilized to perform IHC optimization. All primary antibodies were tested using human tonsil tissue as a positive control. A Leica Bond III automated stainer (Leica Biosystems, Nussloch, Germany) was used to stain for Alpha-SMA, CD44, CD68, and CD163. Briefly, primary antibodies were incubated overnight at 4℃ in a humidified chamber and the respective secondary antibodies conjugated to horseradish peroxidase (Vector Labs, Newark, CA) were added and incubated for 30 minutes at room temperature. The staining was visualized with 3,3’-diaminobenzidine (DAB) HRP substrate kit (Vector Labs, Newark, CA) and counterstained with hematoxylin.

For immunofluorescence (IF) staining, HEI-193 cells were seeded and cultured on glass coverslips for 48 hours before being fixed using 4% PFA. Staining was performed for HAS1 (1:250, Invitrogen PA595599), HAS2 (1:200, Invitrogen PA5115388), HAS3 (1:250, PA589266). Primary antibodies were incubated overnight at 4℃ and the secondary antibody (donkey anti-rabbit, 1:500, AlexaFluoro 594, Invitrogen) was incubated for 1 hour at room temperature.

For IF of the VS tumors (n=4/antibody), the slides were deparaffinized and stained using antibodies against HAS1 (1:200), HAS2 (1:100), and HAS3 (1:200) and appropriate secondaries. Slides were imaged using the Olympus FV3000 confocal system at identical laser settings.

***Quantification of Immunohistochemical Staining***

Whole digital slides were created using Zeiss AxioScan.Z1 slide scanner at 40x magnification and images captured with the Aperio ImageScope v.12 software (Leica Biosystems). For quantification of Hyaluronan, CD44, and the cellular components of the tumor microenvironment (Alpha-SMA, CD68, and CD163), the images were converted to 16-bit contiguous TIFFs and imported into FIJI (NIH ImageJ). The respective IHC staining pixel intensities were then calculated as previously described (Zhang 2023). Briefly, the pixel intensity histogram was calculated through automated color-deconvolution algorithms in FIJI, and the mean number of positive pixels was normalized to the total tumor volume for each biomarker.

***ELISA on Human Primary Tumor Secretions***

Fresh human primary tumor samples were prospectively collected from the operating room following microsurgical resection. Primary tumor cultures were established after tumor samples were placed in DMEM (GIBCO) and incubated in 37C for 72-hours with 5% CO2. Tumor secretions were collected by aspirating conditioned media after 72-hours.

An enzyme-linked immunosorbent assay (ELISAs) for tumor-necrosis factor-alpha (TNF-alpha) (Abcam, Ab181421) was performed according to manufacturer protocols using the secretion-containing condition media.

***Cell Culture***

An immortalized human NF2 vestibular schwannoma cell line (HEI-193, gift from Dr. M. Giovannini, UCLA) and a control wild-type immortalized human Schwann cells (HSC, ATCC) were cultured in complete DMEM supplemented with 10% FBS, 10 mL/L penicillin-streptomycin (Pen Strep), and 10 µM forskolin. HEI-193 cells were plated in a 24-well plate at 500,000 cells per well, allowed to reach confluency in 48-72-hours, and treated with recombinant human TNF-alpha (R&D Systems). for 12 hours. An MTT assay (Abcam, Ab211091) was also performed. Both cell lysates and conditioned media were collected after 12 hours. This experiment was repeated four times independently for each variable.

***qRT-PCR***

RNA was extracted using the Qiagen RNeasy kit, reverse transcribed, and one microliter of cDNA was added to the SYBR Green master mix alongside respective forward and reverse primers. The following primer sequences were used: for *GAPDH*, the forward primer is 5’-GGATTTGGTCGTATTGGG-3’ and the reverse is 5’- GGAAGATGGTGATGGGATT-3’; for *HAS1*, the forward primer is 5’- CAAGATTCTTCAGTCTGGAC-3’ and the reverse is 5’- TAAGAACGAGGAGAAAGCAG-3’; for *HAS2,* the forward primer is 5’- GTCATGTACACAGCCTTCAGAGC-3’ and the reverse is 5’-ACAGATGAGGCTGGGTCAAGCA-3’; and for *HAS3*, the forward is 5’- CTTAAGGGTTGCTTGCTTGC-3’ and the reverse is 5’- GTTCGTGGGAGATGAAGGAA-3’. *GAPDH* and *B2M* were used as housekeeping genes.

***Statistical Analysis***

Statistical analysis of patient demographics was performed with contingency tables analyzed by Chi-Square analysis with Fisher’s exact test using GraphPad Prism 9.5.0 (San Diego, CA, USA), and the statistical analysis of the immunohistochemical staining was also performed using GraphPad Prism 9.5.0. The correlation of biomarker staining to MRE stiffness was done using linear regression. For comparison of means in non-parametric data sets, a Mann-Whitney U-test of rank comparisons were used. For parametric data, a one-tailed student’s t-test was used where a pre-determined hypothesis required it, otherwise the two-tailed variation was used. For multiple comparison of means, a one-way ANOVA test with Bonferroni correction was used. Comparison for single variable prediction of binary stiffness was made with univariate logistic regression analysis (IBM SPSS Statistics, Version 29.0.2.0). Statistical significance was defined as an alpha of 0.05 where applicable.
